# Supplementary material for: Are carbon emissions trading and green financial instruments synergistic? -Comprehensive quantitative research based on content analysis
Source: PLoS One. 2024 Mar 7;19(3):e0298601. doi: 10.1371/journal.pone.0298601 (PMC10919601; doi:10.1371/journal.pone.0298601)
Supplement: S1 Data — (ZIP) [file pone.0298601.s001.zip › Data/地级市Z_F工作报告.zip!/╔╜╢1⁄2/║╩╘≤╩╨/└┤╫╘╣1⁄2╓┌║┼í╛┬φ┐╦╩2╛▌═°í┐_2013.docx]

各位代表:

　　现在，我代表市人民政府向大会作政府工作报告，请予审议，并请市政协委员提出意见。

　　一、2012年工作回顾

　　刚刚过去的一年，全市人民在市委的坚强领导下，深入落实科学发展观，大力实施 “双轮驱动”战略，扎实推进“五大基地一大产业”建设，开拓创新，奋力拼搏，经济社会实现了平稳较快发展。预计实现地区生产总值1770亿元，增长13%；三次产业比调整为13.7∶54.5∶31.8，二三产业比重提高1个百分点；完成公共财政预算收入140.3亿元，增长25.7%；城镇居民人均可支配收入、农民人均纯收入分别达到19240元和8190元，均增长15%以上，主要经济指标增幅继续保持全省和周边九市前列，较好完成了市十八届人大一次会议确定的目标任务。

　　（一）狠抓招商引资和大项目建设，发展后劲进一步增强。坚持市县主要领导带队集中招商与小分队招商相结合，实行市县领导干部包基地、包产业、包重点项目责任制，健全大项目建设月调度、季观摩、年终考核推进机制，招商引资和大项目建设成效显著。引进落地过亿元项目200个，其中过10亿元项目83个；在建亿元以上项目568个，建成投产150个。预计完成固定资产投资682亿元，增长23.5%。利用外资实现新突破，实际到账外资1.65亿美元，增长105%。

　　（二）精心组织经济运行，质量效益同步提高。规模以上工业企业新增286家、达到2405家，预计实现主营业务收入4400亿元、利税580亿元，分别增长29.5%和32%，增幅均居全省第一位；入库税金过千万元企业65家，其中过亿元的11家；主营业务收入过10亿元企业40家，其中过百亿元的4家，东明石化、玉皇化工均可跻身中国企业500强。对外经贸逆势增长，预计完成进出口总额32亿美元，增长16.6%。深入实施“质量兴市”战略，新增中国驰名商标5件、山东省著名商标13件、山东名牌8个。扎实推进金融工作，交通银行菏泽分行、单县村镇银行开业，企业股权场外交易实现零的突破；深化银企对接活动，签约资金到位248.7亿元。金融机构贷款余额1062亿元，比年初增加185亿元；存款余额1606亿元，比年初增加279亿元，其中居民储蓄余额1221亿元，比年初增加236亿元。

　　（三）大力推进城镇建设，城乡面貌明显改观。预计城镇化率达到40%，提高2.5个百分点。深入开展城市发展战略研究，完成了四大片区概念性方案规划、7个县城和30个重点镇总体规划修编。实施城建重点工程180项，其中市区40项，人民路桥、大学路桥、点将台路桥建成通车，市图书馆、胜利广场基本竣工，新建改造道路24条，铺设雨污管网50公里、供热主管网12公里，新增集中供热覆盖面积1080万平方米、卫生保洁面积210万平方米、绿化面积86万平方米。成功入选国家可再生能源建筑应用示范城市、再生资源回收利用体系建设试点城市，顺利通过省级卫生城市复审。积极推进城市综合开发，全市竣工房地产面积1365万平方米，其中市区426万平方米。加快镇村建设，新增小城镇建成区面积11.4平方公里，实施整村改造项目204个，新建农村住房5.4万户；改建农村公路2116公里、窄危桥31座。加快重大基础设施工程建设，德商高速菏鄄段、东新高速东明段、220国道菏郓段和济董路升级改造等16项重点交通工程进展顺利；宝源湖水库即将竣工，洪源水库、刘楼水库开工建设。

（四）深入推进农副产品生产加工基地建设，农村经济全面发展。粮食生产再夺丰收，总产127.7亿斤，实现“九连增”；小麦、棉花最高单产均创全省第一。积极推进农业结构调整，新增牡丹种植15.7万亩、造林16.8万亩，林下畜禽存栏量达到1.3亿头（只）。加快推进农业产业化，新增省级农业产业化龙头企业10家、农民专业合作社800家、农业标准化基地20.7万亩，农作物耕种收综合机械化率提高到82%。深入开展农田水利会战，新增改善有效灌溉面积61.6万亩。

（五）积极推进商贸物流基地建设，服务业稳步发展。全面加强服务业 “四大载体”建设，菏泽义乌商品城、单县万隆国际批发城等一大批项目建成开业，第九届中国林交会现场签约32.9亿元。我市被确定为国家家政服务体系建设试点城市。预计实现社会消费品零售总额893亿元，增长15.2%。加快推动旅游产业发展，我市被命名为“中国牡丹之都”，曹州牡丹园创建为国家AAAA级旅游景区。实现旅游总收入65亿元，增长41.3%。预计完成服务业增加值563亿元，占GDP的比重提高1个百分点。

　　（六）扎实推进自主创新和节能减排，转变发展方式取得新成效。坚持创新驱动，加快发展高新技术产业，中科院过程所菏泽成果转化基地、山东省鲁南药物研究院等研发平台揭牌成立；菏泽金正大被认定为国家火炬计划重点高新技术企业，玉皇化工入选国家创新型试点企业；新增省级企业技术中心8家、工程技术研究中心3家、省级创新型企业6家、省级以上高新技术企业6家。预计实现高新技术产业产值1260亿元，占规模以上工业产值的比重提高到29%，达到全省平均水平。深入开展企业能效对标活动，淘汰高耗能设备1500台（套），累计节能60万吨标准煤。加快土地开发整理，依法查处违法用地，盘活闲置低效用地1万亩。全面做好迎淮核查工作，投入资金29.4亿元，建成治污项目392个；实行“河长”负责制，每天减少直排污水12万吨，三条主要河流水质持续改善。

　　（七）不断加大财政投入，民生保障和社会事业发展取得显著成绩。集中财力办好各项民生实事，完成民生支出195.7亿元，占财政总支出的69.5%，比上年提高2.9个百分点。新增城镇就业10.4万人、农村劳动力转移就业29万人。新增社会保险参保17.7万人次，征缴各类保险费50亿元；城乡居民养老保险实现全覆盖，60岁以上老人按时足额领取了基础养老金。开工建设保障性住房13757套，改造农村危房1.2万户。提高了城乡低保、医疗救助、农村五保供养标准，解决了40万农村人口饮水安全问题。预算内教育经费占财政支出的比重达到21.9%，完成校舍安全工程106万平方米，新增幼儿园209处，新建城区中小学15所，新补充教师1946名；省政府批复菏泽职业学院建院；菏泽学院本科教学工作、家政职业学院人才培养工作通过国家教育机构评估。医药卫生体制改革深入推进，一体化管理的村卫生室全部推行基本药物制度，县乡医疗机构全部实行“先诊疗、后付费”服务模式，新农合参合率达到99.9%。在全省率先完成国有文艺院团改革，元代古沉船保护修复基本完成，新建农村文化大院400个，送戏下乡1380场，送电影下乡7.1万场。我市运动员在伦敦残奥会上取得“一银一铜”好成绩。新增城乡社区122处，便民服务水平进一步提高。充分发挥计划生育利益引导作用，人口自然增长率下降到4.75‰。深入开展军民“双拥共建”，首次被评为“全国双拥模范城”。全面加强安全生产、食品药品监管，扎实开展 “百日会战”活动，社会更加和谐稳定。

　　（八）全面加强政府自身建设，行政效能进一步提高。深入开展 “落实年”和“行政程序年”活动，建立督查联席会议制度，启动政务督查联动机制；深化行政审批制度改革，精简审批事项113项，新的市政务服务中心建成启用。自觉接受人大法律监督、工作监督和政协民主监督，认真办理人大代表议案、建议和政协委员提案。充分发挥市长公开电话、行风热线等便民渠道作用，主动接受社会、群众和新闻监督。严格落实党风廉政建设责任制，强化行政监察和审计监督，政府系统廉政建设不断加强。

　　同时，民族宗教、外事侨务、对台事务、人民武装、防震减灾、广播电视、统计、物价、人防、气象、档案、史志、老龄、妇女儿童和关爱等工作都取得了新成绩。

　　一年来，在国际环境复杂多变、国内经济下行压力较大的不利形势下，全市经济社会发展取得这样的成绩，来之不易。这是市委坚强领导、科学决策的结果，是市人大、市政协有效监督、全力支持的结果，是各民主党派、人民团体及社会各界积极参与、通力协作的结果，是全市上下奋力拼搏、扎实苦干的结果。在此，我代表市人民政府，向全市人民，向各民主党派、工商联、人民团体和社会各界人士，向离退休老领导、老同志，向驻菏部队、武警官兵和中央、省属单位，向所有关心支持菏泽发展的同志们、朋友们，表示崇高的敬意和衷心的感谢！

　　各位代表，我们也清醒地认识到，工作中还存在不少问题和薄弱环节，主要是：大项目、大企业较少，支撑跨越发展能力依然偏弱；资源环境约束加剧，自主创新能力不强，“转调创”任务艰巨；地方财力薄弱，社会保障能力差，一些涉及群众切身利益的突出问题还未得到很好解决，部分群众生活还比较困难；个别干部作风不够扎实，政府推动科学发展的能力和依法行政水平有待进一步提高等。在这里需要特别说明的是，去年《政府工作报告》中承诺的市博物馆、县区“两馆”建设和企业上市目标，由于各种原因未能如期完成。对此，我代表市人民政府，向各位代表并通过你们向全市人民，表示深深的歉意！在今后工作中，我们一定加倍努力，采取更加强有力的措施，全面完成各项目标任务，坚决兑现承诺。

　　二、2013年目标任务和重点工作

　　今年是深入贯彻落实党的十八大精神的开局之年，是实施“十二五”规划承前启后的关键一年，也是加快打造科学发展高地的重要一年。做好今年工作，意义重大。尽管面临的发展环境依然严峻，区域竞争压力不断加大，但机遇大于挑战。我市被国家纳入中原经济区规划，省委、省政府全力支持我市打造科学发展高地，并将出台支持鲁西经济隆起带建设的意见，政策叠加效应逐步显现。特别是去年一年的实践，使我们对打造科学发展高地充满了必胜信心。只要我们负重奋进、扎实苦干，抓住机遇、乘势而上，就一定能够实现全市经济社会科学发展、和谐发展、跨越发展、更好更快发展！

　　今年市政府工作的总体要求是：深入学习贯彻党的十八大精神，以邓小平理论、“三个代表”重要思想、科学发展观为指导，紧紧围绕打造区域科学发展高地、全面建成小康社会目标，以招商引资和大项目建设为抓手，以提高经济增长质量和效益为中心，深入实施“双轮驱动”战略，促进“四化”协调发展，大力推进“五大基地一大产业”建设，着力加强自主创新、发展现代农业、繁荣发展服务业、推进生态文明建设、保障和改善民生，努力建设富裕、和谐、美丽、幸福的新菏泽。

　　全市经济和社会发展的主要预期目标是：地区生产总值增长13%；公共财政预算收入增长16%；规模以上工业增加值增长18%；规模以上固定资产投资增长20%；社会消费品零售总额增长15%；进出口总额增长15%；城镇居民人均可支配收入和农民人均纯收入均增长13%；登记失业率控制在3.8%以内；人口自然增长率控制在6.8‰以内；完成省下达的节能减排任务。

　　围绕实现上述目标，重点抓好以下八项工作：

　　（一）着力抓好招商引资，加快扩大对外开放。对外开放是打造科学发展高地的必然选择和强大动力，必须进一步放宽视野、提升境界，全面提高开放型经济水平。一是强力推进招商引资。依托我市资源优势和产业基础，紧盯世界500强、中国500强，重点引进辐射带动能力强、财政贡献大、吸纳就业多的项目。每个县区至少引进5个过10亿元、20个过亿元项目。进一步拓展招商区域，只要有商机，就主动出击、快速跟进；充分发挥企业主体作用，深入开展对外合资合作；举办好春秋两季投洽会，积极参加高交会、香港和台湾山东周等重大招商活动。加强对欧美、东南亚、日韩等国家的境外招商，实际到账外资增长50%以上。建立项目落地长效机制，强化签约项目跟踪调度，进一步提高落地率。二是加快发展对外贸易。加大外向型重点企业扶持力度，推进出口基地和农产品质量安全示范区建设，扩大优势产品出口；落实鼓励进口政策，抓好紧缺资源、关键设备进口和先进技术引进；大力发展加工贸易，实现进出口总额36亿美元以上。三是加强对外交流合作。发挥市开发区精细化工园、东明石油化工园纳入山东半岛蓝色经济区内陆“联动区”的优势，积极承接发达地区产业转移。鼓励有实力的企业赴海外投资、承包工程和建立生产基地，积极推进境外资源开发和劳务合作。

　　（二）着力抓好大项目和园区建设，加快推进产业集聚发展。大项目建设是实现科学发展、跨越发展的总抓手，以拉长产业链条为重点，努力实现大项目建设和产业集聚发展的新突破。一是加大园区建设力度。突出抓好8个过千亿元、6个过500亿元的产业园区建设，明确园区产业定位，加快配套设施建设，健全服务体系，提升承载能力，确保14个园区实现主营业务收入达到4700亿元以上。制定园区利益分享办法，统筹推进新建大型工业项目、城区搬迁企业、重大技改项目，到相应产业园区落地建设，形成特色鲜明、产业集聚发展的园区建设新格局。完善扶持政策，加快市开发区发展，积极推动晋升为国家级开发区；理顺市高新区管理体制，为创建国家级高新区奠定坚实基础。加大对定陶、鄄城、成武的支持力度，促进县域经济协调发展。二是加快产业基地建设步伐。把基地建设任务，落实到企业，细化到项目，强化调度、考核等推进机制，力争战略性新兴产业、石油化工、煤电化工、农副产品生产加工四个基地主营业务收入增长25%以上。三是全力推进大项目建设。围绕培育百亿级企业、千亿级产业、千亿级园区，抓好重大项目策划储备，确保储备生产性项目600亿元以上；用足用好国家和省支持菏泽发展的政策，争取更多项目纳入国家和省计划。对100个市重点项目，实行市县领导包保责任制，确保年内竣工50个以上。

　　（三）着力抓好骨干企业培育，加快提高工业发展水平。新型工业化是改变欠发达面貌的根本出路和重大战略任务，必须全力攻坚，重点突破。一是加快推动企业做大做强。把培育 “百强企业”作为重要抓手，出台扶持政策，每月在媒体公布纳税排名，充分调动企业家干事创业的积极性、创造性，引导企业加快实施管理创新、技术创新、产品创新，确保新增主营业务收入过500亿元的企业1家、过10亿元的10家。突出抓好企业升级改造，确保5个以上项目列入国家重大专项、50个以上进入省千项重点项目规划，实现技改投入增长20%以上。认真落实扶持政策，促进中小微企业快速发展。确保规模以上工业企业新增200家以上，主营业务收入增长25%以上。深入实施名牌、商标战略，新增山东名牌10个、山东省著名商标6件。积极推进信息化和工业化融合，围绕企业产品研发、生产流程控制等环节，大力推广应用现代信息技术，不断提高自动化、智能化水平。二是切实提升企业自主创新能力。深入实施创新驱动发展战略，安排落实并充分使用好科技创新专项资金，引导全社会加大投入，确保研发投入占GDP的比重达到1%以上。加快市高新技术创业服务中心、市科研成果转化服务中心等研发平台建设，鼓励支持企业开展产学研合作，扎实做好集成创新和引进消化吸收再创新工作。深入推进技术创新提升工程，争取省级以上科技计划35项，实施自主创新重大专项10项，完成发明专利申请660件以上。突出抓好30家重点高新技术企业发展和6个高新技术特色产业园建设，新增省级以上高新技术企业4家，高新技术产业产值占规模以上工业的比重达到30%以上。三是强化运行服务。搞好监测预警分析，及时解决企业生产经营中的关键问题。强化煤电油运等生产要素供给协调，保障企业生产经营需要。建立银政企合作长效机制，鼓励金融机构创新信贷产品和服务模式，新增贷款200亿元以上。积极引进金融机构，大力推进企业上市，进一步拓宽融资渠道。

　　（四）着力抓好城镇建设，加快城乡统筹发展步伐。城镇化是扩大内需的最大潜力，是经济增长的重要引擎，必须切实加快建设步伐，确保城镇化率提高2.5个百分点。一是加快推进规划编制。完成市区总体规划修编、7个县城和30个重点镇控制性详细规划编制、1500个新型农村社区布局规划，进一步提高城乡规划覆盖率。严格规划管理，切实维护规划的权威性、严肃性。二是大力提升承载功能。突出抓好市区基础设施建设，坚持“地上地下”一起抓，打通八一东路，完成淮河路等25条道路建设；加快完善地下管网等配套设施，新铺设雨污管网36公里、供热主管网15公里。加快县城基础设施建设，提升综合服务和承载能力。切实抓好小城镇建设，落实支持30个重点镇建设的各项政策，加快实施“五个一工程”，增强小城镇产业发展、公共服务等功能，吸引更多农民向城镇聚集。三是积极推进城镇综合开发。修订完善房屋征收办法，促进和谐拆迁。加快旧城开发、棚户区改造，市区突出抓好四大片区开发建设，着力打造精品工程。全市竣工房地产面积1450万平方米以上，其中市区460万平方米以上。四是强化城镇管理。推进城市管理创新，完善数字化城市综合管理系统，全面提升市政、市容、交通和物业管理水平。深入推进城市管理重心下移，建立城乡环境综合整治长效机制，巩固提高城乡保洁、绿化、美化、亮化成效。五是加强重大基础设施建设。加快东新高速东明段、德商高速菏鄄段建设，力争220国道、济董路竣工通车，启动105国道、砀定公路菏泽段升级改造工程。力争新万福河、郓城新河航道开工建设。积极做好济菏开城际高铁项目前期工作，力争取得突破性进展。推进现代水网建设，加快洪源水库、刘楼水库建设进度，开工建设箕山河、九女等11座水库。实施坚强智能电网建设工程，新增输电线路513公里、变电容量177万千伏安。

　　（五）着力抓好现代农业发展，加快繁荣农村经济。解决好“三农”问题是全部工作的重中之重，必须毫不放松地抓实抓好，认真落实各项强农惠农富农政策，加大扶贫开发力度，千方百计增加农民收入。一是调整优化农业结构。认真组织实施国家新增千亿斤粮食产能规划，深入开展高产创建活动，力争粮食总产达到130亿斤。优化产业布局，加快牡丹、芦笋、大蒜、山药、中药材等特色产业发展。进一步加大牡丹产业扶持力度，加快推进产品深度研发、龙头企业培育和种植基地建设。加快发展林下养殖，新建示范场区100个。二是创新农业生产经营体制机制。加大农业产业化支撑企业扶持培育力度，新增市级农业产业化龙头企业20家、农民专业合作社400家。着力培育新型生产经营主体，积极扶持联户经营、种养大户、农村经纪人，带动更多农民发家致富。加强农村土地承包经营权流转平台建设，积极推进规模化经营。三是提升农业物质技术装备水平。启动市农产品质量检测中心建设，完善农技推广服务、农产品质量检测和动植物疫病防控三大体系，推动乡镇农业综合服务机构建设，加快构建农业社会化服务新机制。做好科技兴农工作，加快农业科技成果转化和推广。新发展标准化基地20万亩， “三品一标”农产品认证达到200个以上。加大农业机械化推进力度，农作物耕种收综合机械化率达到83%以上。深入开展农田水利会战，新增改善有效灌溉面积80万亩。

　　（六）着力抓好 “四大载体”建设，加快服务业发展步伐。服务业是转方式调结构的重要内容，是城镇化发展的有力支撑和必然要求，必须加快推进 “四大载体”建设，确保服务业增加值占GDP的比重提高1个百分点以上。一是突出抓好商贸物流业。启动菏泽口岸和保税物流中心建设，加快十大物流园区、十大城市综合体、十大专业批发市场建设，提升档次，扩大规模，增强辐射带动能力。健全完善农村商贸流通体系，深入开展“满意消费惠万家”活动。继续办好林产品交易会、农资交易会、房地产博览会等大型展会，促进会展经济快速发展。二是加快发展文化旅游业。充分挖掘“一都四乡”文化潜力，更好地把文化产业发展与旅游景区建设紧密结合起来。围绕8大文化产业发展，突出抓好27个重点项目建设、10大旅游景区开发，启动文化生态保护工程，做好元代古沉船、定陶汉墓的后续保护和开发利用工作。搞好旅游产品开发和精品线路整合，推进与周边地市的互动合作。办好牡丹文化旅游节和全国“十艺节”菏泽分会场各项活动，提高综合效益和对外影响力。旅游总收入增长20%以上，文化旅游产业增加值占GDP比重达到4.5%以上。三是积极发展新兴服务业。认真落实扶持政策，深入推进家政服务体系试点城市建设，积极发展证券、保险等商务服务业，鼓励发展医疗保健、休闲养老等社区服务业，加快发展电子商务、文化创意等现代服务业。

　　（七）着力抓好资源节约和环境保护，加快提升可持续发展能力。良好的生态环境是人民群众的殷切期盼，是政府义不容辞的责任，必须多措并举，全面加强生态文明建设，努力打造生态菏泽、美丽菏泽。一是强力推进节能降耗。完善节能预警调控机制，统筹抓好工业、建筑、交通等各个领域的节能。严格项目节能评估审查、新增能耗等量淘汰制度，从源头上控制能耗过快增长。深入开展企业能效对标活动，淘汰落后高耗能设备1200台（套），节能60万吨标准煤以上。加快推进可再生能源建筑应用示范城市、再生资源回收利用体系试点城市建设，大力发展清洁能源、可再生能源。积极推动政府机关节能，创建节约型公共机构示范单位。二是全面推进资源集约节约利用。严格执行资源利用许可制度，大力推广节水节材新技术，努力降低资源消耗。加快牡丹区可持续发展试验区、东明循环经济示范县、郓城煤化工循环经济示范园建设，发挥好示范引导作用。实行最严格的水资源管理制度，加快节水型社会建设。认真落实耕地保护责任制，严厉查处违法违规用地；严格用地审批管理，进一步提高投资强度、建筑密度；搞好土地综合治理和增减挂钩项目实施，加大闲置低效用地清理整合力度，切实降低亿元GDP土地消耗。三是加大生态环境保护力度。严把项目环评审批关，严格落实“三同时”制度，切实控制新增污染；加强环境执法监管，严厉查处违法排污行为。继续推行“河长制”，进一步完善“治、用、保、防、控”流域治污体系；着力抓好市区垃圾中转站配套建设和曹县、单县污水处理厂扩建，半数以上的建制镇完成污水处理站、垃圾中转站建设。搞好污水管网的铺设和对接，加强治污设施运营管理，提高污水收集率和再生水利用率，切实解决市区和县城建成区生活污水直排问题。进一步做好迎淮核查工作，确保取得优异成绩。狠抓大气污染防治，增加“蓝天白云、繁星朗月”天数。大力推进植树造林，加快黄河故道湿地等水质净化工程建设，新增造林12万亩；加快绿化工程建设，新增城镇绿化700万平方米，争创国家级园林城市。

　　（八）着力抓好民生保障，加快推进和谐社会建设。人民群众对美好生活的向往，就是我们的奋斗目标，必须进一步加大民生投入，让发展成果更多更公平地惠及全市人民，努力建设幸福菏泽。一是切实改善农民生产生活条件。扎实推进农村住房建设与危房改造，新建农房5万户，改造危房1.2万户。加快农村饮水安全工程建设，新增饮水安全人口60万人。提高农村公路通行能力和通达深度，改建农村公路600公里，改造窄危桥30座。二是进一步改善城镇居民生活环境。加快实施安居工程，开工保障性住房10240套。强化供暖供气设施建设，市区新增集中供热覆盖面积300万平方米以上、民用天然气用户3万户；确保春节前完成城区至平泰干线的天然气管道铺设工作，切实解决城区居民供气不足问题。加强城镇卫生保洁，新增保洁面积500万平方米。积极推进客运公交建设，市区新增公交线路4条、新能源汽车50辆。加快菏泽火车站提升改造工程建设，启用站前广场。三是努力扩大就业。加大新增就业和创业扶持力度，多渠道开发就业岗位，扎实做好复员转业军人安置就业、零就业家庭人员就业援助、失业人员再就业、失地农民就业等工作，新增城镇就业9万人以上，农村劳动力转移就业16万人以上。四是健全完善社会保障体系。积极做好城乡养老保险制度衔接工作，继续扩大社会保险覆盖面，新增参保6万人次以上，确保养老金按时足额发放。认真落实社会保障标准增长机制，城市低保月保障标准提高20元，农村低保年保障标准提高200元；城乡医疗平均救助标准均提高500元；农村五保集中和分散供养标准均提高200元；孤儿机构养育和分散养育标准分别提高200元、120元。开工建设市社会福利院，新建县级社会福利中心2处、农村中心敬老院15处。五是大力支持教育发展。切实抓好校舍安全工程，新建改造中小学校舍49万平方米。加快城区学校建设，新建城区中小学12所，逐步解决大班额和择校问题。大力发展学前教育，新增幼儿园200所，完成县区实验幼儿园、乡镇中心幼儿园建设。完善教师增补机制，公开招录1500名中小学、幼儿园教师。积极支持菏泽学院、菏泽医专、菏泽职业学院等院校发展，进一步提高办学水平。六是努力提高人民健康水平。抓好基本药物等重点品种监管，保障群众用药安全。深入推行“先诊疗、后付费”服务模式，方便群众就医。提高新农合保障水平，政府补助标准提高40元。完善重大疾病保障机制，将20种重大疾病纳入医疗保险保障范围。提高基本公共卫生服务均等化水平，免费为城乡居民提供10项基本公共卫生服务。实施妇幼安康工程，全面推行计划生育免费孕前优生健康检查项目。完成单县公立医院综合改革试点。实施市立医院扩建改造、增加病床500张，实施市妇幼保健院迁建，开工建设市二院门诊病房综合楼、市三院门诊楼，启动市中医院二期工程，每个县重点办好1-2所县级医院。启动市体育学校、市全民健身中心、市残疾人康复中心建设，建成县级残疾人康复中心2处。七是不断丰富群众精神文化生活。启用市图书馆，开工建设市规划展览馆、文化艺术馆，加快推进市科技馆前期工作。完成县区文化馆、图书馆主体工程，新建农村文化大院500家，送戏下乡1320场、送电影下乡7万场，新增有线数字电视用户50万户。八是全力维护群众生命财产安全。强化安全监管和责任追究，高度重视交通安全，加强消防指挥体系建设，遏制重特大事故发生。健全社会矛盾纠纷排查化解和突发事件应急救援机制，强化食品安全监管，严厉打击制售假冒伪劣商品行为，努力保障人民群众生命健康安全。完成“天网”工程建设，构建全天候、广覆盖的治安防控网络，严厉打击“两抢一盗”等违法犯罪行为，切实提高群众的安全感和满意度。

　　三、切实加强自身建设

　　各位代表，时代的新要求、发展的新重担、人民的新期盼，对政府工作提出了新的更高要求。我们一定牢记全心全意为人民服务的宗旨，锤炼求真务实的优良作风，打造优质高效的政务服务，筑牢拒腐防变的思想底线，始终做到为民、务实、清廉，努力建设尽责有为、人民满意的政府。

　　（一）大力改进工作作风。严格落实中央“八项规定”和省委“十条实施办法”，认真按照市委高境界、高标准、高效率、高效益工作要求，扎实开展“作风改进年”活动，以转变作风的实际成效取信于民。坚持理论联系实际、学以致用的学风，大力加强学习研究，全面提高把握规律、破解难题、推动工作的能力。倡导严谨细致、扎实苦干的工作作风，坚持工作重心下移，更多地深入基层、深入一线、深入群众开展调查研究，认真解决群众关注的实际问题，现场为企业为群众办实事、解难题。大力压缩会议、精简文件，提倡开短会、讲短话、讲实话，坚决改变以会议贯彻会议、以文件落实文件的简单化工作方式。坚持目标管理和过程管理并重，进一步加强政务督查，完善督查联动机制，确保各项工作落到实处，取得实实在在的成效。

　　（二）强力推进依法行政。加强政府法制建设，严格按照法定权限、法定程序履行职责、行使职权。强化行政监察，严肃查处各种行政不作为、乱作为、慢作为行为。扎实推进行政审批制度改革，加强电子政务建设和政务服务中心管理，全面提升行政效能。充分发挥政府网站和市长公开电话作用，进一步推进政务公开。自觉接受人大及其常委会的法律监督、工作监督和政协的民主监督，认真听取民主党派、人民团体意见，广泛接受社会公众和新闻舆论监督，确保权力在阳光下运行。

　　（三）努力打造清廉政府。严格遵守廉洁从政的各项规定，认真落实党风廉政建设责任制，切实把廉政建设的要求落实到政府工作的各个领域、各个环节，做到干部清正、政府清廉、政风清明。强化审计监督和执法检查，深入开展纠风和专项治理，大力整治部门、行业不正之风，坚决查处各种违法违纪案件。厉行节约，反对浪费，严控 “三公经费”支出，集中财力保民生、促发展。始终把人民置于心中最高位置，常怀敬畏之心，多谋为民之策，恪尽职守，夙夜在公，树立廉洁勤政的良好形象。

　　各位代表，打造科学发展高地的良好开局，使我们信心满怀；全面建成小康社会的美好前景，使我们干劲倍增。让我们在市委的坚强领导下，深入贯彻党的十八大精神，凝聚力量，扎实苦干，攻坚克难，全力加快科学发展步伐，为建设幸福美好的新菏泽而努力奋斗！
